# Solution Synthesis of Actinide Chalcogenide and Oxychalcogenide Nanoparticles

**DOI:** 10.1002/anie.6508645

**Published:** 2026-06-30

**Authors:** Cheyenne Orozco, Orlando C. Stewart, Nicole Vanagas, Greg Morrison, Hans‐Conrad zur Loye, Hope E. Smith, Karah Knope, Sarah L. Stoll

**Affiliations:** ^1^ Department of Chemistry Georgetown University Washington DC USA; ^2^ Department of Chemistry University of South Carolina Columbia South Carolina USA

**Keywords:** nanoparticles, oxychalcogenide, uranium

## Abstract

We report the synthesis and characterization of a series: UOS, UOSe and β‐US_2_, the first examples of uranium‐chalcogen‐containing nanomaterials using colloidal synthetic techniques. The challenge to these syntheses is the oxophilicity of uranium, as uranium dioxide is difficult to avoid and the only actinide nanomaterial reported previously. All reagents, the metal, chalcogen source and solvent, were investigated to understand phase formation in solution. We found a post‐synthetic modification, commonly known as digestive ripening, that led to size control of the β‐US_2_ nanoparticles. The phases were confirmed using powder x‐ray diffraction and Rietveld analysis, as well as microscopy ( transmission electron microscopy), and magnetic susceptibility measurements. The oxychalcogenide nanomaterials are antiferromagnetic, in contrast to previously reported UO_2_ nanoparticles. As found in bulk β‐US_2_, the nanoparticles are paramagnetic, with a moment consistent with localized f electrons.

## Graphical abstract

We report the synthesis of UOS, UOSe, and US_2_ nanoparticles under colloidal conditions. The magnetic properties were measured of all three materials and compared to prior studies of UO_2_ nanoparticles. Nanoparticle phases were determined using powder diffraction, and size by transmission electron microscopy.

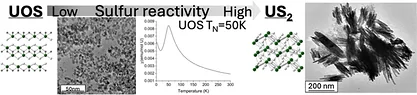

## 1 Introduction

Studies of covalency in actinide compounds have attracted interest for practical reasons (to develop separation methods that reduce radioactivity in waste), as well as for a fundamental understanding of 5f orbitals [1]. The 5f and 6d orbitals are closer in energy to the valence orbitals of S than O, contributing the degeneracy driven covalency in uranium chalcogenides [2], and the soft polarizability of chalcogens has been proposed to enhance this effect [3]. As a result, efforts to probe uranium covalency has motivated the syntheses of molecular complexes supported by chalcogen donors [4, 5]. Similarly, the synthesis of new uranium chalcogenide materials [6] reflects a parallel effort to elucidate the nature of bonding, and electronic structure [1], where in the solid state, covalency influences the density of states band dispersion [7]. Actinide materials often exhibit a ‘dual nature’ of the 5f orbitals, where 5f electrons can be localized (narrow bands) or itinerant (dispersed, partially filled band). The degree of localization is critical to understanding properties, in particular superconductivity [8], and magnetism [9]. One of the motivations for investigating nanomaterials was to develop a framework for how electronic states evolve from molecules to materials. The study of nanomaterials thus forms a bridge between molecular and solid‐state properties [10]. Until now, reports of nanoscale actinide chalcogenides are absent.

An important reason for the lack of uranium chalcogenide nanoparticle studies is a synthetic problem commonly confronted for uranium, suitable starting reagents. For molecular chemistry, there is a paucity of commercially available salts, and even simple uranium halides, which are poorly soluble in nonpolar solvents, require preparation [11]. One common approach to improve solubility in nonpolar solvents used in nanoparticle synthesis and avoid oxygen donors, is to employ silyl‐amides [12]. However, U[N(SiMe_3_)_2_]_4_, an ideal and soluble U(IV) precursor, is formed in low yields and requires the synthesis of U[N(SiMe_3_)_2_]_3_ first and then an oxidation step to U(IV) [13]. Even simple salt elimination reactions have found limited success in expanding the coordination chemistry of uranium to S and Se donor ligands [5]. In the solid‐state, the problem of uranium oxophilicity can lead to impurities, inspiring the development of a synthetic approach known as the ‘boron chalcogen method’ [6]. The role of the boron is to sponge up oxygen, leaving the chalcogen to form compounds with uranium.

It is not surprising that the first efforts to synthesize actinide containing nanoparticles have focused on UO_2_ [14]. The advantage of thermodynamic stability makes UO_2_ synthetically more accessible, and accounts for its use in nuclear fuel. Oxygen‐donating ligands, such as carboxylate or beta‐diketonate ligands are common in nanoparticle synthesis and it has been reported that either U(IV) (e.g., U(acac)_4_) or U(VI) (UO_2_(acac)_2•2_H_2_O)) are successful reagents for UO_2_ nanoparticles [15]. Careful solution conditions have been developed for size control over the UO_2_ nanoparticles [15], while shape control was recently demonstrated using surfactant concentration [16] and modeled based on ligand binding energies and surface oxidation conditions [17].

From the viable options for metal precursors, we investigated the use of uranium tetrachloride, UCl_4_, and developed several reproducible nanoparticle syntheses of the magnetic oxychalcogenides, UOS, and UOSe, as well as the dichalcogenide US_2_. The uranium oxychalcogenides are antiferromagnetic and provide an interesting comparison to prior magnetic studies of UO_2_ nanoparticles [18]. To obtain pure uranium disulfide nanoparticles required rigorous purification of reagents, highly reactive sulfide sources, and careful reaction conditions. We were able to synthesize US_2_, as the beta polymorph, which had a needle‐like, agglomerated morphology. Despite the high demonstrated oxophilicity of uranium, it was possible to use oleic acid as a digestive ripening solvent post‐synthetically to produce monodispersed small spherical nanoparticles of preformed β‐ US_2_.

## 2 Results and Discussion

The lack of reports of uranium chalcogenide nanoparticle syntheses leaves open an opportunity to interrogate phase stabilization from solution. The lower reaction temperatures of colloidal synthesis can allow for kinetically stabilized phases [19]. Uranium favors lower oxidation states when paired with soft chalcogens (Q = S, Se, and Te), where U(III) is observed in UQ and U_2_Q_3_, whereas U(IV) is found in UQ_2_ and UQ_3_ (Q = S, Se, and Te) [20]. Phase selectivity is an important challenge for nanoparticle synthesis, particularly for polychalcogenides which can exhibit chalcogen–chalcogen bonding, or metals that are redox active with complex phase diagrams [21, 22]. For transition metal sulfides, the more ‘reactive’ the chalcogen (often correlated with the bond dissociation energy of S in the reagent) [23, 24], the more chalcogen‐rich phase is stabilized [25]. Tuning the reactivity of sulfur reagents is one approach to phase control in nanoparticle synthesis, allowing access to many compositions of nanoscale binary metal sulfide materials [23, 26].

**FIGURE 1:**
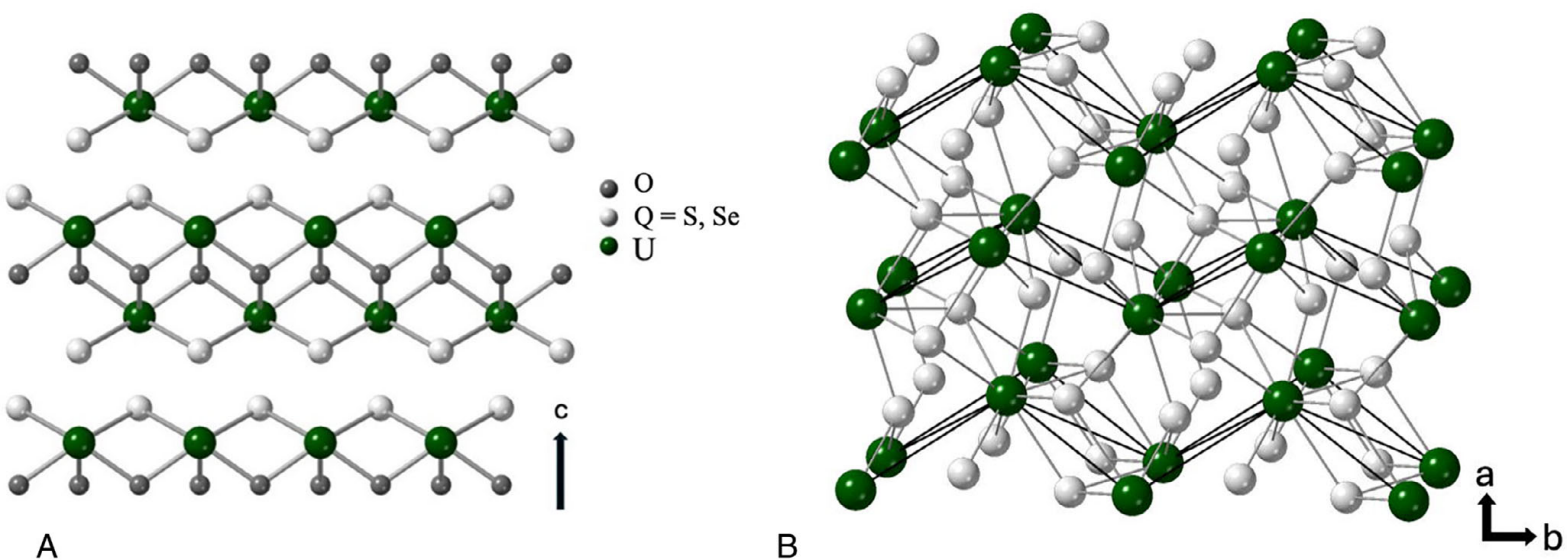
Structure of UOQ (Q = S, Se) (a) and β‐US_2_ (b). Green atom (U), dark grey (O), and white (Q).

The synthesis of uranium oxychalcogenides (UOQ, Q = S, Se) nanoparticles underscores the strong oxophilicity of uranium, as the conditions were air‐free with no intentional oxygen reagent. The oxychalcogenides are isostructural to NpOS [27], which adopts the tetragonal PbFCl‐type structure, composed of [Q‐(U‐O)‐(O‐U)‐Q] layers, where the interlayer interactions decrease with increasing Q radii (Figure 1a) [28]. Within each layer, uranium preferentially coordinates oxygen, while the softer chalcogen resides at the terminal positions. The structural anisotropy increases from Q = S, Se, Te to *c/a* >2 for UOTe, an antiferromagnetic van der Waals material [29]. van der Waals magnetic materials are of current interest for understanding magnetism in two dimensions.

Bulk UOQ materials have been reported to be electronically small‐gap semiconductors (< 1 eV) [30], and antiferromagnetic with similar spin structure (++–) for both Q = S, Se [31, 32]. By contrast, the UQ monochalcogenides are ferromagnetic [33]. The disulfides have only one magnetic polymorph (ferromagnetic γ‐US_2_), while all polymorphs of USe_2_ are antiferromagnetic [34]. The room temperature polymorph β‐US_2_ adopts a PbCl_2_‐related structure with two distinct sulfur sites and nine‐coordinate uranium (Figure 1b) [35]. Both β‐US_2_ and UTe_2_, have been described as ‘nearly ferromagnetic’, and UTe_2_ exhibits novel superconductivity [36, 37].

### 2.1 Synthesis of UOQ (Q = S, Se)

Our reactants were chosen from common semiconductor nanoparticle syntheses with the exclusion of oxygen‐containing reagents [38]. We selected UCl_4_ because it avoids oxygen donors and can be prepared relatively easily from uranium oxides. The nanoparticle syntheses described here used UCl_4_, with Tri‐OctylPhosphine Sulfide or TOPS, which functions both as a surfactant/solvent and chalcogen transfer reagent [39]. At the start of this investigation, every reaction produced UO_2_ without an obvious source of oxygen (Figure S1). After testing alternative surfactants and chalcogen reagents, we believed the source was from minor impurities in UCl_4_ synthesized from hexachloropropene and UO_3_•2H_2_O [40]. To eliminate these impurities, an alternative synthesis using chemical vapor transport was adopted [41], which produced a more pure and anhydrous source of single crystals of UCl_4_ (PXRD in Figure S2). We found this uranium chloride when reacted with TOPS, and alternatively TOPSe, formed UOS or UOSe oxychalcogenides.

**FIGURE 2:**
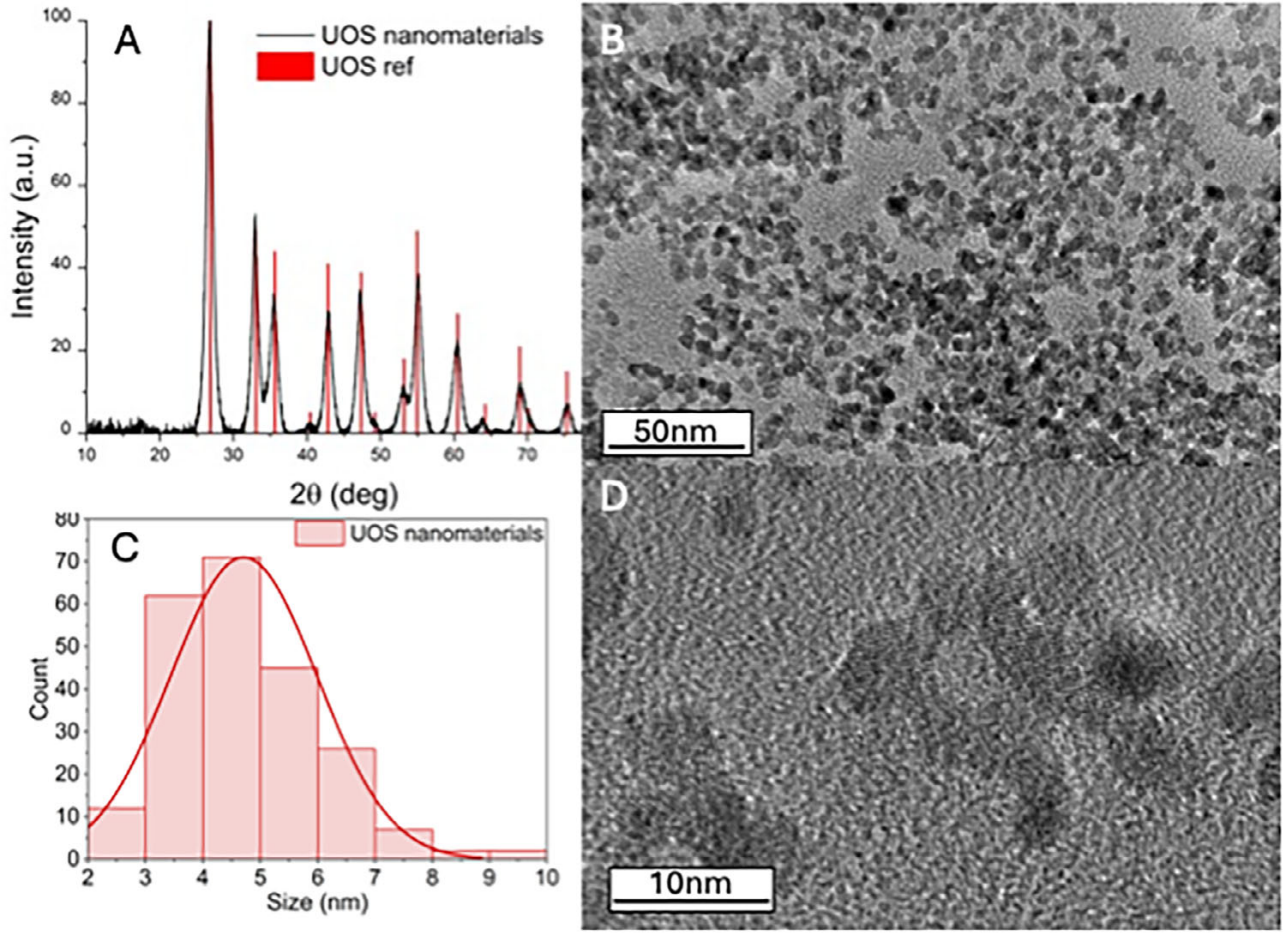
PXRD with reference PDF 00‐004‐0527 (A) TEM (B) and histogram with average diameter of 4.7 ± 1.3 nm, (C) HRTEM, and (D) of UOS nanoparticles.

Although the oxychalcogenides are a step closer to the goal, the source of oxygen remained a question and confirms that even with quite pure UCl_4_, the synthesis is sensitive to the chalcogen and solvent (surfactant). The phase purity of UOS nanoparticles was evidenced by PXRD shown in Figure 2. It has long been observed that the surfactants used in nanoparticle synthesis often contain small amounts of highly active contaminants that can affect reproducibility [42]. We believe the oxygen incorporation is due to our solvent, TOP. Secondary phosphines were among the first active impurities found in TOP [43], and can affect the reactivity of chalcogens but an unlikely source of oxygen. Tri‐OctylPhosphine is air‐sensitive and can form TOPO, and TOPO has long been known to contain phosphinate and phosphinic acid impurities [44]. However, we believe octanol, which has only recently been reported in TOP as a functional impurity [45], acts as the relevant oxygen source here.

The UOS nanoparticles are small and spherical, the average size was 4.7 (± 1.3) nm with a narrow size distribution seen in transition electron microscopy (TEM) studies and histogram (Figure 2, and summary of particle size determination in Table S1). Generally, nanoparticles synthesized with TOP as the capping ligand tend toward small and spherical. The small size of the UOS nanocrystals is also evident in the GSAS refinement, which found an average particle size of 9.1 nm (± 0.1 nm). We believe the size based on PXRD is more representative of the sample average, given the relatively lower statistics of TEM measurements. There were many nanoparticles that appear to be single crystalline from high‐resolution TEM (HRTEM). The FFT analysis reveals d‐spacings that align with the crystal structure of UOS. The yield of the UOS nanoparticles appears to be limited by the poor solubility of UCl_4_ in TOPS.

**FIGURE 3:**
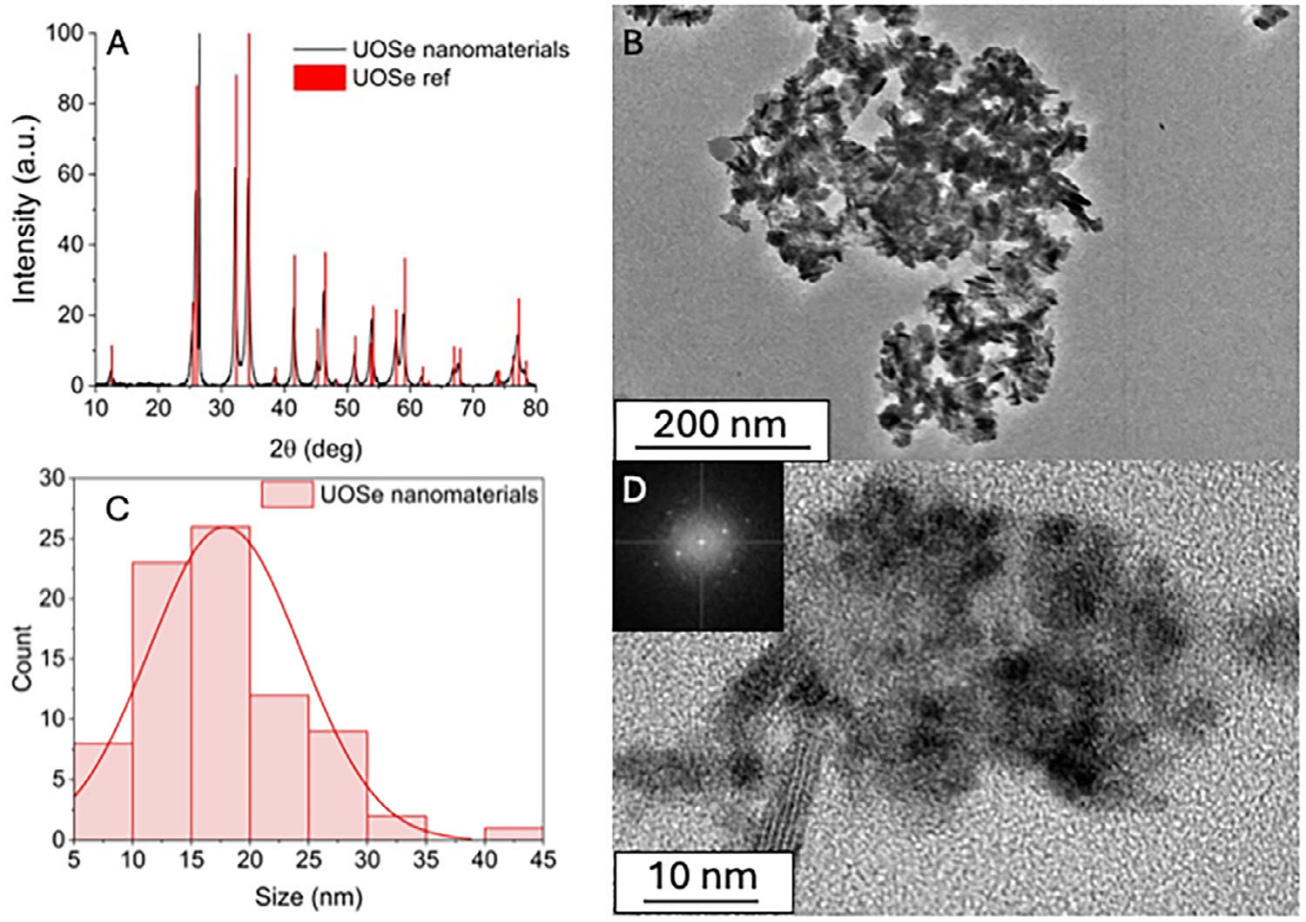
PXRD with reference PDF 01‐078‐8378 (A) TEM, (B) histogram with average diameter of 17 ± 6.4 nm, (C) HRTEM, and (D) of UOSe nanoparticles.

Sulfur transfer from TOPS is slow and mediated through a phosphorous to metal exchange, limiting the concentration of sulfur [24]. Previously, it has been observed that reagent ‘reactivity’ for the same molecular structure of R‐Q (e.g., R = tri‐methylsilyl or R = tri‐butylphosphine) increases down the series Q = S<Se<Te [46]. Given the enhanced reactivity from S to Se, we were interested in whether TOPSe would lead to a higher chalcogen content. However, the pure oxyselenide product, UOSe, was observed in the powder diffraction (Figure 3), confirming the oxophilicity of uranium dominates even with more reactive chalcogen reagents. Based on TEM, the UOSe nanoparticles were larger (17 ± 6.4 nm) and more agglomerated, consistent with increased precursor reactivity (comparison to UOS in Table S1). The GSAS refinement suggested some anisotropy, with the long axis 29.2 ± 0.1 nm, and 7.6 ± 0.1 nm. Collectively, these results indicate that slow chalcogen transfer and solvent oxygen impurities lead to UOQ phases.

### 2.2 Magnetism of UOS and UOSe

**FIGURE 4:**
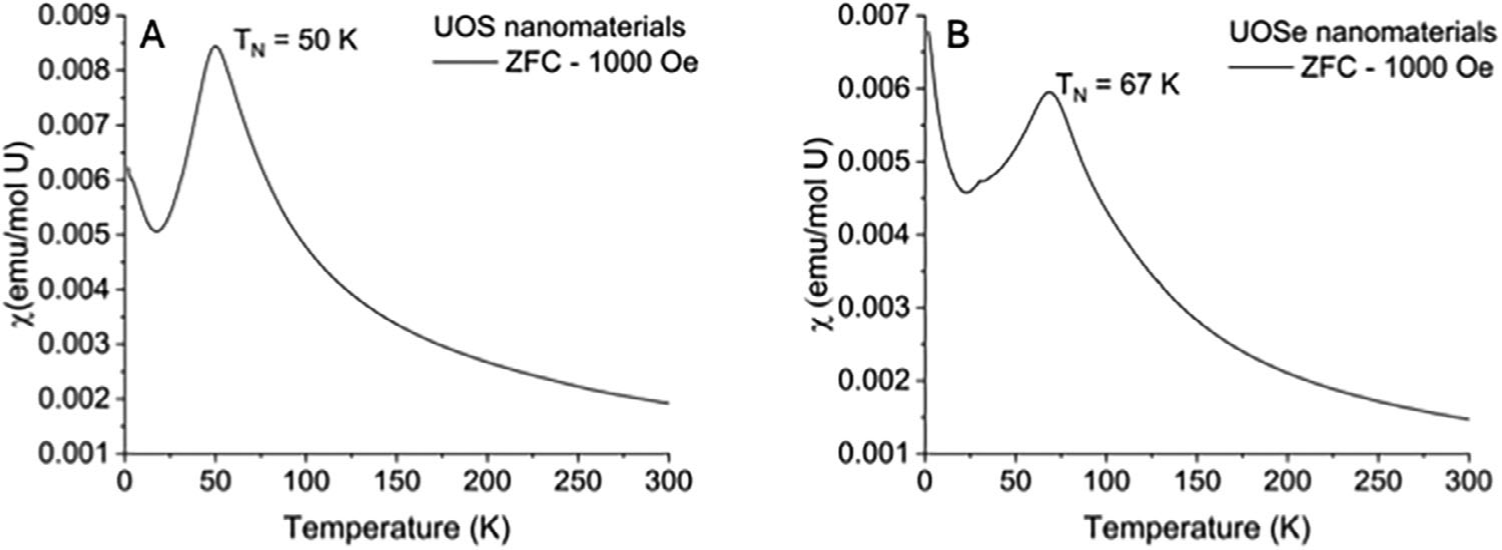
The χ(T) for UOS nanoparticles (4A) and UOSe (4B), taken at 1000 Oe.

The magnetic properties were of interest to provide a handle for interpreting the extent of 5f orbital localization. The magnetic behavior of uranium materials tends to correlate with the U···U distance, where separations > 3.5 Å favor magnetic ordering (Hill criterion) [32]. The model is that shorter U···U distances tend to result in itinerate 5f states due to direct 5f overlap that lead to wide energy bands, whereas greater distances lead to more localized, highly coupled 5f electrons (in narrow bands) [47]. Magnetic measurements provide insight into the extent of moment localization (magnitude of the µ_eff_), as well as the type of magnetic ordering. The moment is calculated from the high‐temperature 1/χ‐vs‐T data, Curie–Weiss plots [48, 49]. Delocalization can lead to a reduction in magnitude, although for this purpose neutron studies are more accurate [50]. A peak in the χ(T) is indicative of antiferromagnetic ordering as can be seen in the graphs in Figure 4.

There are several features of interest in this data. For UOS (Figure 4A), the Néel temperature of 50K and (*µ*_eff_ 2.41, *Θ* = −64) compares well with the bulk 55K (*µ*_eff_ = 2.2, *Θ* = −108) with Curie–Weiss fits in Figure S3 [51]. A comparison of UOS and UO_2_ nanoparticle magnetism raises some intriguing questions. Uranium dioxide has a different structure type (fluorite) but is also antiferromagnetic in the bulk with a Néel temperature of 31K [52], and arguably more ionic (i.e., more narrow, localized DOS). Nanoparticles of UO_2_ exhibited no peak in the χ(T) data, although the Weiss constant (Θ ∼ ‐31K) was negative, consistent with antiferromagnetism [18]. However, the moment was also quite low, µ_eff_ = 0.61 [18], which could be due to a nondegenerate ground state, or itineracy, or a change in oxidation state. Notably, these values are substantially reduced from those found in the bulk (*µ*_eff_ = 3.2, *Θ* = −220K). The conclusion was that size reduction of UO_2_ repressed magnetic order. In the bulk, oxygen hyper‐stoichiometry (UO_2+x_) is known to decrease T_N_ and significantly reduce the moment due to oxidation, which provides an alternate hypothesis [53].

The magnetic susceptibility of UOSe has a similar χ(T) curve to UOS, with a peak at 65 K (Figure 4B). When comparing the Curie–Weiss fits (Figure S4), the nanoparticles of UOSe have a *µ*_eff_ of 2.08 and *Θ* = −49.6, which are both lower than observed for bulk powders (*µ*_eff_ = 3.3, and *Θ* = −120) [53]. Unfortunately, in the bulk there is wide variability in the Néel temperature reported for UOSe; from 68 K (from specific heat measurements) [32]. to 74K (magnetic susceptibility of powders) [54], and 90 K (from resistivity measurements) [55]. Measurements of single crystals of UOSe observed a peak as high as 100 K perpendicular to the layers but a flat χ(T) parallel to the layers, with a moment of 2.2 BM [32]. There are a range of possible reasons for the reduced Néel temperature of the UOSe nanoparticles, including non‐stoichiometry and anisotropy. We note a small bump in the χ(T) of UOSe, which occurs at 29K, which we believe could be due to UO_2_. There is no evidence in the PXRD of this impurity but would note that UOSe is far more air‐sensitive than the UOS, and there is no deviation in the UOS magnetic data.

### 2.3 Synthesis and Characterization of β‐US_2_

**FIGURE 5:**
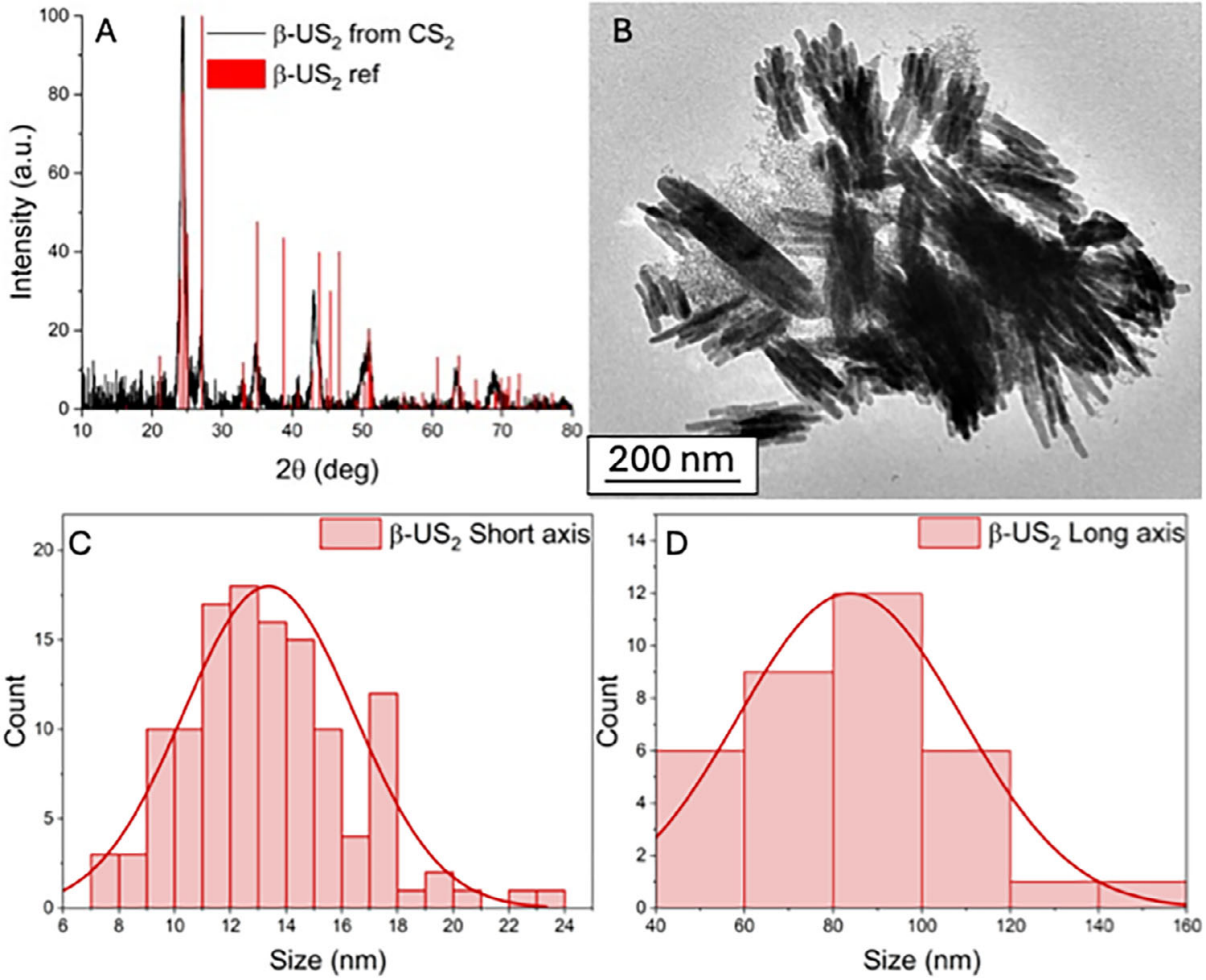
PXRD of β‐US_2_ from CS_2_ with reference PDF 04‐002‐6779 (A) TEM, (B) histograms from (C, D) TEM data.

To suppress oxygen incorporation and access sulfur‐rich phases, we modified the synthesis to increase the ‘reactivity’ of the metal and chalcogen source. The term reactivity is employed because the active solution species can be elusive to measure in situ. One approach to increasing metal reactivity is to add LiHMDS (Li hexamethyldisilyzane, LiN(Si(CH_3_)_2_)_2_) [56]. It has been reported that ‘amide assisted’ nanoparticle synthesis enhances metal reactivity via two potential reactions. One reaction is direct metathesis with metal halides to form M[NSiMe_3_)_2_]_n_, and the other is deprotonation of oleylamine to form a M(NHR)_x_ complex [57]. This led us to shift from TOP to oleylamine as the solvent, which can to act as a reducing agent, a potential advantage for favoring the lower uranium valence [58]. As discussed in the introduction, previous literature points to the instability of U[NSiMe_3_)_2_]_4_, so the U(NHR)_4_ complex seems more likely. For greater chalcogen reactivity we turned to carbon disulfide, which can produce metal dichalcogenides, MS_2_, from metal chlorides in oleylamine for a range of early transition metals, M [59]. It is possible that carbon disulfide could form a carbamate, that chelates to the metal before forming the metal sulfide. Using this combination of reagents, we were successful in synthesizing a phase‐pure uranium chalcogenide, US_2_, which was identified from powder diffraction to be the most stable polymorph, β‐US_2_, Figure 5. The formation of the beta polymorph is consistent with the calculated Energy Above the convex Hull (EAH) of 0, according to Materials Project [60].

Evidence suggests that CS_2_ in oleylamine can generate H_2_S in situ, an intermediate also observed when elemental sulfur is dissolved in oleylamine [57]. To test whether the active species was H_2_S, as the critical reagent, and to avoid the use of a volatile precursor, we also reacted S_8_ in oleylamine with UCl_4_ and LiHMDS, and again obtained a pure uranium sulfide, β‐US_2_ based on PXRD (products from both reactions are compared in Figure S5). The combination of LiHMDS and sulfur in oleylamine has been studied in detail for nanoparticle synthesis, and it has been proposed that elemental sulfur reacts both with oleylamine and LiHMDS, and that nanoparticles do not form without the LiHMDS [61]. To understand the role of LiHMDS, as a set of control reactions we used both chalcogen precursors without LiHMDS. Interestingly, there was no difference in phase or morphology of the β‐US_2_ from CS_2_ (Figure S6), but for the reaction with sulfur, we discovered that UOS formed instead (Figure S7). Although we had considered each reagent separately, as frequently found the reagents interact and cannot be considered in isolation.

A report by Macdonald [25], investigating phase control of iron sulfide nanoparticles has some interesting comparisons for the work discussed here. In this study, the reaction of the metal halide (FeCl_2_) in oleylamine with a relatively unreactive sulfur reagent (Ph_2_S), led to metal oxide (Fe_3_O_4_) product. Interestingly, the disulfide (FeS_2_) only formed with the combination of highly reactive sulfur reagents (e.g., di‐allyl‐disulfide) and oleylamine as the solvent. The combination of highly reactive sulfur reagents in octadecene, by contrast also formed metal oxides. The alkylamine was argued to play a critical role in stabilizing the sulfur anion in solution, so that the reactivity of the sulfur reagent and the solvent are important. We diluted olelyamine with the non‐coordinating solvent, octadecene, in an effort to inhibit agglomeration. However, any degree of dilution led to amorphous materials. After thorough screening of a range of reaction conditions (see Table S2), we were able to conclude that the high reactivity of the sulfur precursor in oleylamine, in combination with carefully treated starting materials leads to pure uranium chalcogenides.

**FIGURE 6:**
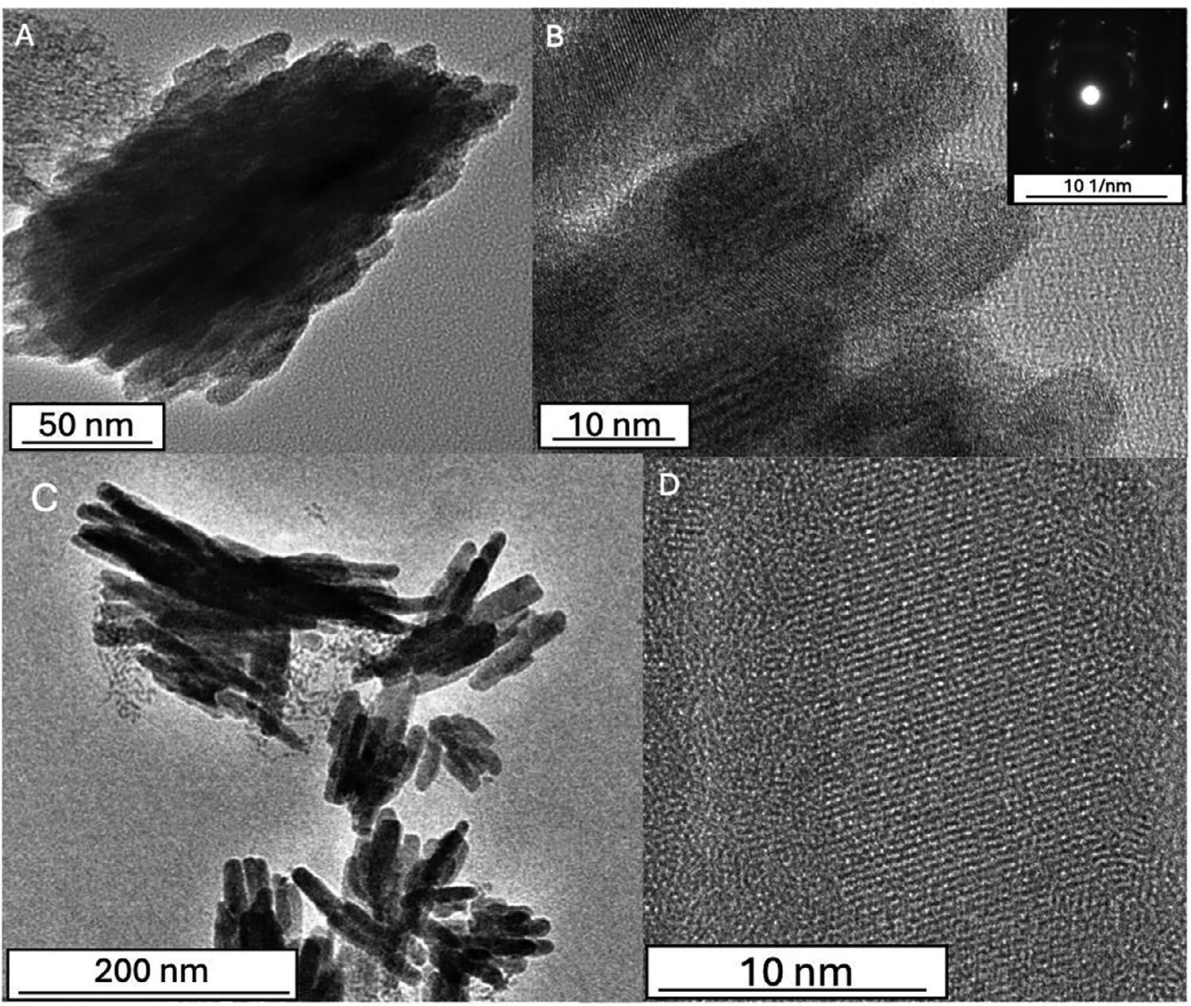
Comparison of β‐US_2_ from UCl_4_, LiHMDS and two sulfur sources: S_8_ (top row, A,B) and CS_2_ (bottom row, C,D).

The morphology of the β−US_2_ is quite distinct from the UOS or UOSe, in forming highly associated anisotropic nanocrystals, which appear to be rod‐like. Both the polymorph, and the nanocrystal shape appear to be governed by thermodynamics. The equilibrium shape of a crystal is dominated by the facets with lowest surface energy (according to the Wulf construction) [62]. Here the nanocrystals have the same needle‐shaped morphology exhibited by bulk β‐US_2_ single crystals [36]. The difference between nanoparticles synthesized from CS_2_ and elemental sulfur was most apparent in the morphology of the nanoparticles (Figure 6). Although the β‐US_2_ from CS_2_ was agglomerated, HRTEM images (Figure 6D) suggests the anisotropic nanocrystals are single crystalline. Based on TEM, the CS_2_ material has differentiated rods with a short axis of 13 ± 3 nm x 83 ± 25 nm. Using GSAS to refine the material, the aspect ratio was similar (12 × 111 nm). By contrast the material from elemental sulfur has highly intertwined regions of polycrystalline material (Figure 6B), perhaps suggestive of an oriented attachment of nanocrystals. What is interesting is that not only are nanorods hard to identify in the TEM of the material made by S_8_, the GSAS refinement fit better assuming isotropic materials, with a dimension of 13.2 ± 9 nm. Although both S_8_ and CS_2_ generate H_2_S in OLA, S_8_ is thought to produce radicals that induce surface corrosion and morphological disorder [57].

We did not consider extending this approach to USe_2_, as CSe_2_ is toxic, and Se does not dissolve in oleylamine [63], so we believe this class of materials will require a new synthetic approach.

### 2.4 Digestive Ripening of β‐Us_2_

Synthetic efforts to control particle size and monodispersity are key to studying size dependent properties. The primary approach to control semiconductor nanoparticle diameters is to use time as the variable. The most common nanoparticle growth mechanism, Ostwald ripening, describes a process where large particles grow and smaller particles dissolve over time, leading to an increase in average diameter and dispersion of sizes over time. Recognizing that the strength of the metal–ligand interaction has an influence on overall particle size, a post‐synthetic step has been identified that modifies the ligand surface and leads to size focusing [64]. This method, called ‘digestive ripening’ is often considered the reverse of Ostwald ripening. In digestive ripening, the large particles are etched, and deposition occurs on smaller nanoparticles, leading to a uniform size. There is a thermodynamic explanation in which the final size reflects a balance of the nanoparticle system (e.g., surface energy) such that for a given capping ligand (digestive ripening agent), there is an equilibrium size attained [65]. There is also a kinetic argument, that etching and dissolution are balanced in rate by a redeposition process resulting in a particular diameter [66].

**FIGURE 7:**
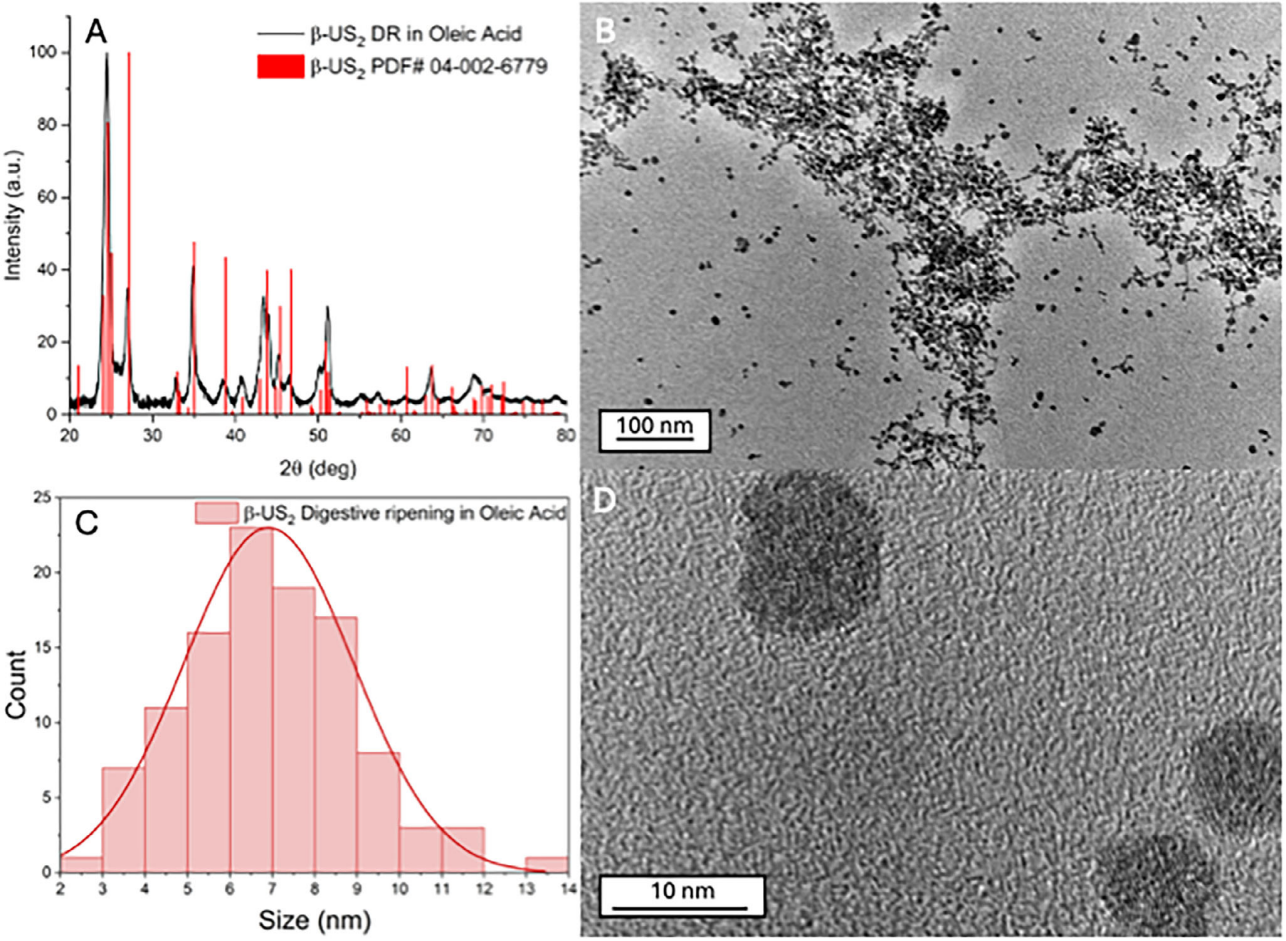
Digestive ripening of β‐US_2_ (synthesized from LiHMDS and S_8_) in oleic acid. (A) PXRD of β‐US_2_ with reference PDF 04‐002‐6779. (B) TEM, (C) histogram, and (D) HRTEM.

To achieve digestive ripening, the success in part depends on the coordinating ability of the new solvent to act as a ligand [62]. Despite the sensitivity of uranium to oxygen‐containing reagents in the synthesis, oleic acid was selected due to the strong coordinating ability of the carboxylate for uranium. However, we found that there were distinct differences in whether we used the β‐US_2_ from CS_2_ (the anisotropic rods) or β‐US_2_ from S_8_ (more agglomerated and isotropic). Using the β‐US_2_ nanorods from CS_2_ and heating at low temperatures in oleic acid appeared to have very little effect on the morphology. The effect of oleic acid on the more crystalline anisotropic material made from CS_2_ was unchanged, we believed due to the single crystalline nature of the nanorods (TEM data in Figure S8 compares the β‐US_2_ from CS_2_ before and after digestive ripening). This was in distinct contrast to preformed β‐US_2_ from S_8_ (more isotropic based on GSAS refinement) in oleic acid. At a low temperature in oleic acid we observed a reduction in particle size dispersions but no evidence of oxidation in our PXRD (Figure 8). Interestingly, the morphology transformed from the highly agglomerated material (which refined to 13.2 ± 0.9 nm) to nearly spherical small particles that GSAS refinement found to be 9 ± 0.1 nm. Histograms of the TEM were slightly lower, ∼ 7 ± 2 nm but reasonably separated monodispersed diameters (Figure 7B–D). The data suggest a model in which the oleic acid is able to etch the less crystalline material between attached nanospheres of β‐US_2_ synthesized from S_8_, leading to smaller, more spherical product. While this may exceed the definition of digestive ripening, we continue to describe the materials this way, as we do observe size focusing and reduced particle dimensions.

### 2.5 Magnetic Characterization of β‐Us_2_

The β‐US_2_ has been reported to undergo a metal‐insulator transition, with local 5f electrons at low temperature and delocalized electrons at high temperatures based on neutron scattering experiments [48]. DFT calculations suggest the band gap is ∼0.9 eV [67], close to experimental measurements (resistivity found an E_g_ ∼1 eV). The nanomaterials were black, as expected for such a small band gap material. The report of magnetic susceptibility measurements on bulk powders found a µ_eff_ of 3, but the 1/χ data exhibited a positive deviation from CW behavior [35]. Single crystals exhibit highly anisotropic magnetism, with no clear ordering temperature but along the 001 axis the µ_eff_ was 3.4 and the Θ ∼8K. The 010 and 100 were more similar with a µ_eff_ above 3, but Θ ranging from −93 K to −130 K [36].

We were interested to see whether the µ_eff_ would change with a decrease in particle size, so we compared the β‐US_2_ as synthesized to the digestively ripened material (Curie–Weiss fits in Figures S9 and S10). For the β‐US_2_ used in digestive ripening, the µ_eff_ slightly increased (from 2.22 BM to 2.64 BM) with a decrease in particle size (13 to 9 nm). It is interesting to note that the β‐US_2_ synthesized from CS_2_, which was even larger (12 × 111 nm) had an even lower moment (1.70 BM, in Figure S11). There are other important factors that could affect the moment, such as surface oxidation. However, the trend is unusual (increasing moment with decreasing size) because typically the smallest nanoparticles have the highest surface area so most oxidation and lowest moment. None of the samples exhibit a clear ordering temperature in the χ(T) data, consistent with the lack of ordering found in bulk β‐US_2_. In addition, neither UOS or UO_2_, potential oxidation products were observed in the susceptibility near 50 or 30 K, respectively. The preparation of a larger range of sizes with additional characterization methods to probe questions of stoichiometry and surface oxidation are in progress. For this work, the increase in moment for the smallest material is consistent with our conclusion from PXRD that digestive ripening did not lead to measurable nanoparticle oxidation.

## 3 Conclusion

The syntheses described here are consistent with efforts in molecular and solid‐state synthesis of uranium chalcogenides in that the reagents and conditions need to be carefully controlled to prevent the formation of uranium oxides. As a metal reagent UCl_4_ can be prepared as anhydrous crystals using chemical vapor transport, and while the reactivity of the chalcogen can be tuned to control phase, solvent impurities represent an additional hurdle. We have established phase control to prepare both UOS and UOSe nanoparticles which exhibit properties like the bulk material, including effective moments ∼2 BM, and Néel temperatures readily related to the bulk material. The first uranium chalcogenide nanoparticles were prepared by tuning the reactivity of the metal source and the chalcogen and switching the solvent away from the phosphine surfactant. Despite the sensitivity of these syntheses to oxygen, it was possible to use oleic acid to digestively ripen the β‐US_2_, and control particle size with no evidence of oxygen incorporation into the nanomaterial.

## Author Contributions

**Cheyenne Orozco**: conceptualization, writing – original draft, writing – review and editing, formal analysis, investigation, data curation. **Orlando C. Stewart, Jr**: conceptualization, data curation, formal analysis. **Nicole Vanagas**: investigation, resources. **Greg Morrison**: data curation. **Hans‐Conrad zur Loye**: writing – review and editing. **Hope E. Smith**: data curation, investigation. **Karah Knope**: conceptualization, data curation, writing – review and editing. **Sarah L. Stoll**: conceptualization, investigation, funding acquisition, writing – original draft, methodology, validation, visualization, formal analysis, project administration, supervision, resources.

## Conflicts of Interest

The authors declare no conflicts of interest.

## Supporting information




**Supporting File**: anie73243‐sup‐0001‐SuppMat.docx.

## Data Availability

The data that support the findings of this study are available on request from the corresponding author. The data are not publicly available due to privacy or ethical restrictions.
