# Supplementary material for: Solution Synthesis of Actinide Chalcogenide and Oxychalcogenide Nanoparticles
Source: Angew Chem Int Ed Engl. 2026 Jun 30;65(36):e6508645. doi: 10.1002/anie.6508645 (PMC13528546; doi:10.1002/anie.6508645)
Supplement: Supplementary file 1 [file ANIE-65-e6508645-s001.docx]

**Supporting Information**

Solution synthesis of actinide chalcogenide and oxychalcogenide nanoparticles

Cheyenne Orozco,^[a]^ Orlando C. Stewart, Jr.,^[a]^ Nicole Vanagas,^[a]^ Greg Morrison,^[b]^ Hans-Conrad zur Loye,^[b]^ Hope E. Smith,^[b]^ Karah Knope,^[a]^ Sarah L. Stoll*^[a]^

[a] Department of Chemistry
Georgetown University
37^th^ and O Sts, NW, Washington DC, 20057

[b] Department of Chemistry
University of South Carolina
631 Sumter St., Columbia, SC 29208

**Experimental section**…………………………………………………………………………2-3

**Figures**

Figure S1. Representative PXRD of experimental UO_2_.……………………….…………..….4

Figure S2. PXRD of UCl_4_ from chemical vapor transport (photo of crysals)……………..…..4

Figure S3. Curie Weiss fit for UOS nanomaterials…………………………..…………..….….4

Figure S4. Curie Weiss fit for UOSe nanomaterials…………………………………………...5

Figure S5. Comparison of PXRD of β-US_2_ nanoparticles, from CS_2_ and S_8_...………….…......5

Figure S6. TEM images of β-US_2_ synthesized from CS_2_ with and without LiHMDS …..….....5

Figure S7. PXRD of UOS synthesized from S_8_ and oleylamine without LiHMDS ………........6

Figure S8. TEM images of β-US_2_ from CS_2_ before and after ‘digestive ripening’………….....6

Figure S9. Curie Weiss fit for β-US_2_ synthesized with sulfur and OLA………….…………....6

Figure S10. Curie Weiss fit for β-US_2_ after digestive ripening…………………...……………7

Figure S11. Curie Weiss fit for β-US_2_ synthesized from CS_2_.…………………...…………….7

Table S1. Summary of nanoparticle sizes from TEM, GSAS………………………………….8

Table S2. Summary of reaction conditions for β-US_2_ synthesis……………………………….8

Table S3. Summary of magnetic data……………………………………………………...…..9

Experimental

Methods and Materials

**Caution!** The uranium in the syntheses reported here contains U-238 from depleted uranium. Depleted uranium is an α emitter and standard procedures for radioactive material handling should be followed. All radioactive materials were handled in laboratories designated for radioactive materials.

Uranium dioxide was obtained from International Bio-Analytical Industries, Inc. and annealed under H_2_/N_2_ (5%) at 800°C for three days and stored under a nitrogen atmosphere prior to use. Anhydrous aluminum trichloride (AlCl_3_, 99.99%), sulfur (S_8_, 99.998%), selenium (Se, -100 mesh, 99.99%), lithium hexamethyldisilazane [Li(HMDS), 99.99%], carbon disulfide (CS_2_, ReagentPlus®, ≥99.9%) and anhydrous trioctylphosphine (TOP, >97%) were purchased from Sigma Aldrich, stored in a nitrogen glovebox and used as received. Toluene was purchased from Sigma Aldrich and used as received. Oleic acid is purchased from Sigma Aldrich and degassed by heating to 100°C under vacuum for a minimum of 2 hours prior to use. Hexanes and 200 proof ethanol were purchased from Fischer Scientific and used as received. Oleylamine, 98% was purchased from Sigma Aldrich and purified by vacuum distillation followed by degassing under vacuum at 100°C and stored in a nitrogen glovebox.

Powder X-Ray Diffraction (PXRD) patterns were collected on a Rigaku Ultima IV diffractometer equipped with a highspeed DTEX silicon-strip detector using Cu Kα radiation. Air-free PXRD samples are prepared under nitrogen and covered with Magic® Scotch Tape prior to removal from the glovebox. Samples for transmission electron microscopy (TEM) and scanning electron microscopy (SEM) were prepared by sonicating particles in toluene and drop casting on amorphous Formvar carbon-coated Cu grids. High-resolution TEM (HRTEM) and energy-dispersive X-ray spectroscopy (EDS) analyses were performed on a JEOL JEM-2100F FEG TEM operated at 200 kV at the Advanced Imaging and Microscopy Lab at University of Maryland. SEM images were collected on a Zeiss SUPRA55-VP. Rietveld refinements were computed using GSAS-II. Magnetic property measurements were collected on a Quantum Design MPMS 3 SQUID magnetometer. Zero-field cooled magnetic susceptibility measurements were performed from 2 to 300 K in an applied field of 0.1 T. The raw data were corrected for sample holder and radial offset and sample shape according to the method described in the literature (see reference 48), and diamagnetic Pascal’s constants (reference 49).

Synthesis of UCl_4_ precursor

The method was adapted from Rudel and Kraus’s uranium halide syntheses via chemical vapor transport.^43^ UO_2_ (2.70 g, 0.0100 mol) and AlCl_3_ (3.0g, 0.0225 mol) are added to a two-chamber quartz ampule then fitted with a gas inlet adaptor. The ampoule is transferred to a Schlenk line, cooled under liquid nitrogen then vacuum is applied for approximately 30 minutes. The ampoule is then flame-sealed while holding vacuum. To create the temperature gradient, the reactant-containing “hot” end of the ampoule is wrapped in Fisherbrand™ Samox™ heavy insulated cloth heating tape and heated to 350°C while the “cool” end is wrapped with a separate cloth heating tape and heated to 215°C. The vapor transport is allowed to take place over four days after which the reaction is allowed to cool to room temperature radiatively. The ampoule is opened in a nitrogen glovebox and the resultant UCl_4_ is stored at -20°C under nitrogen atmosphere.

Typical nanoparticle synthesis

All nanoparticle syntheses were prepared in an MBraun glovebox under an atmosphere of nitrogen. Reactions were prepared in round bottomed flasks and fitted with a condenser and gas adapter before attaching to a Schlenk line where the reaction was conducted under Ar atmosphere following standard Schlenk techniques.

Synthesis of TOP-Q (Q=S, Se)

Under a nitrogen environment, trioctylphosphine chalcogenide is prepared by stirring elemental chalcogen (10 mmol) in anhydrous trioctylphosphine (10 mL) until it dissolves to a clear solution.

Synthesis of UOQ nanomaterials

In a typical synthesis, the TOP-Q (10 mL of TOPS or 20 mL of TOPSe) and uranium tetrachloride (200mg, 0.527mmol) is added to a round bottomed flask then moved to a Schlenk line. The reaction mixture was degassed under vacuum by heating to 120°C for approximately 20 minutes. After degassing, argon was flushed through the apparatus and the temperature of the solution was increased to 330°C then held at constant temperature for an hour. The reaction was then allowed to cool to room temperature. The reaction was then opened to air and transferred to a centrifuge tube with hexanes (~5 mL) and precipitated with ethanol (~35 mL) via centrifugation for 10 min at 1500 rpm. The supernatant is disposed, and the remaining precipitate was redispersed in hexanes and precipitated with ethanol as described before. This cycle was repeated. The final product was dispersed in hexanes and stored at -20°C.

Synthesis of β-US_2_ nanomaterials from elemental sulfur

In a typical synthesis, in a round bottom flask charged with a stir bar, sulfur (32.0 mg, 1.00 mmol) is dissolved in oleylamine (6 mL) affording a red-orange solution. UCl_4_ (100mg, 0.263 mmol) and LiHMDS (160mg, 0.956 mmol) are added and the reaction is moved to a Schlenk line. The solution is degassed at 100°C for approximately 20 minutes then flushed with Argon and heated to 330°C and held at constant temperature for an hour. The reaction was then allowed to cool to room temperature radiatively and opened to air for nanoparticle isolation. The reaction mixture is transferred to a centrifuge tube with hexanes (~5 mL) and toluene (~5 mL). The solution is then precipitated with ethanol (~35mL) via centrifugation for 5 minutes at 1500 rpm. The supernatant is disposed, and the remaining powder is redispersed in hexanes and precipitated following the previously described procedure. The final product is dispersed in hexanes and stored at -20°C.

Synthesis of β-US_2_ nanomaterials from carbon disulfide

In a typical synthesis, in a round bottomed flask, UCl_4_ (50 mg, 0.13 mmol) is added to oleylamine (6 mL) before CS_2_ is added (0.40 mL, mmol). The reaction is transferred to a Schlenk line, and the solution is degassed at room temperature for approximately 20 minutes then flushed with Argon and heated to 300°C and held at constant temperature for an hour. The remainder of the nanoparticle work-up is identical to the previously described procedure.

*Digestive Ripening of β-US_2_ nanomaterials*

In a Schlenk flask, 7 mL oleic acid is added and 2mL of previously synthesized β-US_2_ nanomaterials suspended in hexanes is added. The solution is heated to 80°C under vacuum for one hour to evacuate the hexanes. The solution is then heated to 120°C and the temperature is maintained for two hours. Afterwards, it is cooled and the nanoparticle work-up is completed identical to the previously described procedure.

**Figure S1**. Representative PXRD of experimental UO_2_. Reference pattern PDF #04-003-4848.

**Figure S2.** PXRD of UCl_4_ precursor synthesized via chemical vapor transport (left). (Star indicates peak from Scotch Tape used for air-free data collection). Photo of UCl_4_ crystals (right).

**Figure S3.** Curie Weiss fit for UOS nanomaterials.

**Figure S4.** Curie Weiss fit for UOSe nanomaterials.

**Figure S5.** Comparison of PXRD of β-US_2_ nanoparticles synthesized from CS_2_ and S_8_. Reference pattern PDF #04-002-6779.

**Figure S6**. TEM images of β-US_2_ synthesized from CS_2_ with LiHMDS (left) and without LiHMDS (right).

**Figure S7**. PXRD of UOS synthesized from elemental sulfur and oleylamine without LiHMDS. Reference pattern PDF #00-004-0527.

**Figure S8.** TEM images of β-US_2_ from CS_2_ before and after ‘digestive ripening’

**Figure S9.** Curie Weiss fit for β-US_2_ synthesized with sulfur and OLA.

**Figure S10.** Curie Weiss fit for β-US_2_ after digestive ripening.

**Figure S11.** Curie Weiss fit for β-US_2_ synthesized from CS_2_.

**Table S1.** Summary of nanoparticle sizes from TEM, GSAS.

Table S2. Summary of reaction conditions for β-US_2_ synthesis

| **Solvent** | **Sulfur Source** | **Additive** | **Product** |
| --- | --- | --- | --- |
| OLA | CS_2_ | LiHMDS | β-US_2_ |
| OLA | CS_2_ | - | β-US_2_ |
| OLA | S_8_ | LiHMDS | β-US_2_ |
| OLA | S_8_ | - | UOS |
| OLA + ocatedecene | S_8_ | LiHMDS | amorphous |

**Table S3.** Summary of magnetic data. References (1=51), (2=55) (3=36).

| **Materials** | **Ordering temp (K)** | **μ_eff_ (μ_B_)** | **θ_P_ (K)** | **R^2^ for CW fit** |
| --- | --- | --- | --- | --- |
| nano UOS | 50 | 2.41 | -63.6 | 0.9997 |
| bulk UOS^1^ | 55 | 2.24 | -108 | - |
| nano UOSe | 67 | 2.08 | -49.6 | 0.9993 |
| bulk UOSe^2^ | 100 | 3.30 | -120 | - |
| β-US_2_ from S+OLA | - | 2.22 | -90.3 | 0.9964 |
| β-US_2_ from DR | - | 2.64 | -57.7 | 0.9974 |
| β-US_2_ from CS_2_ | - | 1.70 | -162.0 | 0.9901 |
| bulk β-US_2_^3^ | - | 3.1 – 3.5 | 8 (H∥[001])  -93 (H∥[100])  -130 (H∥[010]) | - |
